# Randomised controlled trials of mood stabilisers for people with autism spectrum disorder: systematic review and meta-analysis

**DOI:** 10.1192/bjo.2022.18

**Published:** 2022-02-24

**Authors:** Bharati Limbu, Shoumitro Deb, Meera Roy, Rachel Lee, Ashok Roy, Oluwafemi Taiwo

**Affiliations:** Research Assistant, Department of Brain Sciences, Faculty of Medicine, Imperial College London, UK; Visiting Professor of Neuropsychiatry, Department of Brain Sciences, Faculty of Medicine, Imperial College London, UK; Honorary Consultant Psychiatrist, Hereford and Worcestershire Health and Care Trust, UK; Specialty Registrar in Psychiatry of Intellectual Disabilities, Coventry and Warwickshire Partnership NHS Foundation Trust, UK; Honorary Professorial Fellow, Warwick Medical School, University of Warwick, UK; Core Trainee in Psychiatry, Coventry and Warwickshire Partnership NHS Foundation Trust, UK.

**Keywords:** Autism spectrum disorder, mood stabilisers, anti-epileptics, RCTs, systematic review

## Abstract

**Background:**

Despite the widespread use of psychotropic medications in people with autism spectrum disorder (ASD), there is limited evidence to suggest that psychotropic medications including mood stabilisers are effective in individuals with ASD.

**Aims:**

To carry out a systematic review and meta-analysis of randomised controlled trials (RCTs) that assessed the effectiveness of mood stabilisers in people with ASD.

**Method:**

We searched the following databases: Cochrane Library, MEDLINE, Embase, CINAHL, PsycINFO, ERIC, DARE, and ClinicalTrials.gov. In addition, we hand-searched 12 relevant journals. We used the Cochrane Risk of Bias and Jadad scores to assess the quality of included RCTs. We carried out a meta-analysis using a random-effects model.

**Results:**

We included eight RCTs (four on valproate, two on levetiracetam, and one each on lamotrigine and topiramate) that included a total of 310 people with ASD, primarily children. Outcomes were based on core and associated ASD symptoms including irritability and aggression but not bipolar disorder. Only two small studies (25%) from the same group showed definite superiority over placebo and one over psychoeducation alone. Meta-analysis of pooled data on the Aberrant Behaviour Checklist-irritability, Clinical Global Impression Scale-improvement, and Overt Aggression Scale (OAS)/OAS-modified did not show any significant inter-group difference. The rates of adverse effects did not show any significant inter-group difference.

**Conclusions:**

Given the methodological flaws in the included studies and the contradictory findings, it is difficult to draw any definitive conclusion about the effectiveness of mood stabilisers to treat either ASD core symptoms or associated behaviours. Robust large-scale RCTs are needed in the future to address this issue.

PROSPERO registration: CRD42021255467 on 18 May 2021.

Autism spectrum disorder (ASD) is a neurodevelopmental disorder (NDD) that starts in early childhood and often continues into adulthood.^1^ The disorder is characterised by (a) persistent deficits in social communication and social interaction across multiple contexts and (b) restricted, repetitive patterns of behaviour, interests or activities.^1^ The condition affects 1 in 160 of the population.^2^ Comorbidities (overall 70%) including other NDDs such as intellectual developmental disabilities (IDD) (38%) and attention deficit hyperactivity disorder (ADHD) (25–28%), and psychiatric disorders such as psychosis (4–12%), anxiety (18–20%) and depression (11–19%) are much more prevalent in people with ASD compared with the general population.^3–5^ Similarly, the use of psychotropic medication, particularly antipsychotics, psychostimulants and antidepressants, is widespread in this population and seems to have increased in the past decade.^6–9^ However, a recent systematic review and meta-analysis that investigated the effectiveness of anti-anxiety and antidepressant medications in the ASD population did not find any randomised controlled trial (RCT) that used anti-anxiety medications and antidepressants for the treatment of depression and anxiety in the ASD population.^10^ In addition, this meta-analysis did not find any definitive evidence of the efficacy of antidepressants and anti-anxiety medications in improving ASD core symptoms such as language impairment and repetitive behaviours or associated behaviours such as irritability and aggression.^10^ There is weak and indefinite evidence based on small studies that anti-epileptics may be effective in treating patients with personality disorders, conduct disorders or ASD, and psychiatric out-patients.^11–13^ Valproate, however, is contraindicated in women of childbearing age and also in patients with dementia.^14,15^ Hirota and colleagues’ systematic review and meta-analysis, which is the only one we have found on the subject, did not find any significant inter-group difference between anti-epileptic medications and placebo in children and adolescents with ASD.^16^ As this review is now 7 years old, we decided to update the evidence by extending the remit to people with ASD of all ages and including RCTs on all mood stabilisers including anti-epileptic medications to include any studies published since the systematic review by Hirota and colleagues.

## Method

This study was registered with the International Prospective Register of Systematic Reviews (PROSPERO; identifier CRD42021255467) on 18 May 2021.

### Search strategy

We followed PROSPERO guidelines and the Preferred Reporting Items for Systematic Review and Meta-Analysis Protocol (PRISMA-P) checklist to develop our protocol and search strategy. English articles published between January 1985 and June 2021 were searched in the following databases: Cochrane Library, MEDLINE, Embase, CINAHL, PsycINFO, ERIC, DARE, and ClinicalTrials.gov. Relevant journals in the field of ASD (*Journal of Autism and Developmental Disorders*, *Autism Research*, *Journal of Autism Spectrum Disorder*), psychopharmacology (*Psychopharmacology*, *Neuropsychopharmacology*, *International Journal of Neuropsychopharmacology*, *Journal of Clinical Psychopharmacology*, *Human Psychopharmacology*, *Journal of Child and Adolescent Psychopharmacology*), and IDD (*Journal of Intellectual Disability Research*, *Journal of Applied Research in Intellectual Disability*, *Research in Developmental Disability*) were searched for relevant articles published between January 2000 and June 2021. Bibliographies of identified articles were also searched. The search terms used are described in Supplementary Appendix 1 available at https://doi.org/10.1192/bjo.2022.18.

### Selection criteria

The search strategy for the definitive systematic review was finalised following a scoping search. After the initial systematic review search, articles on non-human subjects and duplicates were removed. The titles, abstracts and full articles were screened independently by two authors (M.R. and A.R.) to identify potential articles for inclusion, using pre-piloted eligibility criteria (Supplementary Appendix 2) designed as per Cochrane Library guidelines.^17^ All RCTs in ASD involving mood stabilisers were included. Two authors (M.R. and A.R.) were blind to each other's selection. Any discrepancy was resolved by discussion. The third author (S.D.) did not need to arbitrate because of the full consensus between the two authors (M.R. and A.R.) at the end.

### Participants

All participants had a diagnosis of ASD, defined using standardised or unstandardised criteria, or based on a clinical assessment. No age limit was applied. Studies that included people with IDD were included if the participants also had a confirmed diagnosis of ASD. Only RCTs with more than ten participants were included.

### Intervention

Mood stabilisers including lithium and anti-epileptic medications were included in the study. Other classes of psychotropic medications were excluded.

### Design and comparators

Only RCTs that compared the effectiveness of mood stabiliser medications with a placebo or another form of intervention, e.g. another medication or a psychoeducation programme (PEP) were included. Non-RCT studies were excluded as they are likely to produce a bias.

### Outcome measures

Any standardised validated outcome measures to assess core symptoms of ASD such as language and communication impairment and restrictive repetitive behaviours, any other associated behaviours such as agitation, aggression, irritability, hyperactivity, etc., and symptoms of any psychiatric disorder such as bipolar disorder were included.

### Data extraction

Data were independently extracted by two authors (R.L. and O.T.) using a data extraction *pro forma* modified from a Cochrane template (Supplementary Appendix 3).^18^ Data extraction started on 12 July 2021. Mendeley^19^ was used to organise data from the included articles. A third author (S.D.) acted as an arbitrator if there was any major discrepancy in extracted data by R.L. and O.T. The quality of the included papers was assessed independently by R.L. and O.T. using the Cochrane risk-of-bias tool^20^ (Supplementary Appendix 4) and the Jadad score.^21^ Disagreements were resolved by discussion between the two authors with the third author (S.D.) acting as an arbitrator if necessary. Data on adverse effects were also extracted using the same data extraction *pro forma*.

### Data synthesis

Findings were presented using a narrative format (Table 1). Where it was possible to pool data from more than one study, a meta-analysis was carried out. A meta-analysis using random-effects odds ratios or standardised mean difference (SMD) with 95% confidence intervals was performed depending on the type of data gathered. Heterogeneity was tested using the χ² test and I² statistic test of heterogeneity. If there was substantial heterogeneity (I² > 50%), a further sensitivity analysis was carried out as per the Cochrane guide.^22^ One author (B.L.) approached authors of five included studies asking for missing data, but as none of them responded, data from these RCTs on the Overt Aggression Scale (OAS)/OAS-modified (OAS-M) and Clinical Global Impression Scale-improvement (CGI-I) could not be pooled for meta-analysis.^23–27^

**Table 1:** Summary of findings

Study author(s) and date	Study design, (methods used for ASD diagnosis)	Dose of medications	Participants (*N*, age, (mean ± s.d.), gender, IQ (mean ± s.d.), intervention, FU)	Outcome measures used	Findings, Jadad score	Comments
Divalproex sodium						
Hollander et al (2006)^24^	Parallel-design, double-blind, placebo-controlled, DSM-IV, ADI-R, ADOS	Divalproex sodium: mean dose at end-point was 822.92 ± 326.21 mg/day (range 500–1500/day). Mean trough serum level of divalproex at end-point was 58.23 ± 21.63 μg/ml	Divalproex: 9 PBO: 4 Age: 9.5 (5–17) years (one adult, age 40 years) Gender: not specified IQ: Mean: 60 (range 30–104) FU: 8 weeks	C-YBOCS	Statistically significant improvement in the treatment group compared with PBO in C-YBOCS score at FU (*P* = 0.037). Effect size (d = 1.61) Jadad = 4	Very small sample size and short study duration. Also, *P*-value was 0.05 when data on one adult participant were excluded
Hollander et al (2010)^25^	Parallel-design double-blind placebo-controlled, DSM-IV-TR, ADI-R, ADOS-G. CGI-S score of at least 4 to justify exposure to the medication.	Divalproex sodium <40 kg: 125 mg/day to 250 mg twice daily >40 kg: 250 mg/day to 500 mg twice daily	Divalproex: 16 PBO: 11 Age: 9.66 years (intervention), 8.97 years (control) (5–17 years) Gender: intervention group, 13 boys, three girls; PBO, ten boys, one girl IQ: 52.92 (Range 30–89) FU: 12 weeks	CGI-I, ABC, OAS-M, CYBOCS, VABS, YMRS	Overall, 62.5% in the divalproex group compared with 9% in the PBO group were responders (CGI-irritability OR: 16.7, Fisher's exact *P* = 0.008). A marginally statistically significant improvement was also noted on the ABC-irritability subscale (*P* = 0.048). There was a trend for responders to have higher valproate blood levels compared with non-responders. Participants with an abnormal EEG may have responded better to divalproex than those without any abnormality, but no definitive conclusion could be drawn because of the very small number involved. No statistically significant inter-group differences in either the VABS communication and socialisation domains, or the YMRS scores. Controlling for IQ did not change these findings. No significant inter-group difference on the aggression score according to OAS-M or repetitiveness score as per C-YBOCS. Jadad = 4	Small sample size and short study duration. Also, contradictory findings depending on the outcome measures used; whereas the primary outcome measure showed a significant difference, four secondary outcome measures failed to do so.
Martsenkovsky (2014)^27^	Non inferiority parallel-design double-blind RCT, DSM-IV, ADI-R, ADOS	Divalproex sodium dispensed in the form of sprinkles and titrated to tolerance or serum valproate level between 50 and 100 pg/ml *v*. risperidone (0.01–0.07 mg/kg/day).	Divalproex: 43 Risperidone: 43 Age: 4.87 ± 0.41 years Gender: Dvalporex 27 boys, 16 girls; Risperidone 31 boys, 12 girls IQ: Not specified FU: 16 weeks	CGI-I, OAS-M, ABC	Risperidone was significantly better than divalproex sodium in improving aggression, impulsivity, hyperactivity/non-compliance and stereotypy; CGI-I-irritability (*P* = 0.002), OAS-M (*P* = 0.005), ABC-I (teacher rating) (*P* = 0.04). Jadad = 0	No information on the drop-out rate or inclusion/exclusion criteria
Lamotrigine						
Belsito et al (2001)^23^	Parallel-design double-blind placebo-controlled, ADI-R	Lamotrigine twice daily dose (0.5–5 mg/kg/day)	Lamotrigine: 14 PBO: 14 Age: 5.8 ± 1.75 years; (3–11 years) Gender: 27 boys, one girl IQ: Not specified FU: 18 weeks	AUBC, ABC-C, VABS, PL-ADOS, ADOS, CARS	No significant inter-group difference in scores on the following outcome measures: AUBC, ABC-C, VABS communication or daily living domains, PL-ADOS and CARS. No improvement in stereotypies, lethargy, irritability, hyperactivity, emotional reciprocity, sharing pleasures, language and communication, socialisation, and daily living skills. Marginally significant improvement in VABS social domain (*P* = 0.045). Scores improved by 3.9% for lamotrigine and 0.2% for placebo. Jadad = 5	Unnecessary use of too many outcome measures and a large placebo effect
Levetiracetam						
Wang et al (2017)^33^	Single-blind parallel-design non-placebo-controlled, DSM-5	Levetiracetam 60 mg/kg/day	Levetiracetam + psychoeducation: 32 Psychoeducation alone: 35 Age: 61.6 ± 13.8 months (combined group), 63.1 ± 12.7 months (control group) Gender: 57 boys, ten girls IQ: Not specified FU: 6 months	PEP-3, CVP, EL, RL, CCS, CARS, ABC/AUBC	Both groups showed significant improvements in their behavioural and cognitive functions at FU according to PEP-3, CARS and ABC scores, but all these outcomes were significantly better in the combined than the control group at FU (*P* < 0.05). Subclinical EEG abnormalities improved significantly in the combined (75%) compared with the control group (14.3%) (*P* < 0.05). Jadad = 4	Single-blind and not placebo-controlled
Wasserman et al (2006)^26^	Parallel-design double-blind placebo-controlled, DSM-IV, ADI-R, ADOS	Levetiracetam: 125 mg/day to 250 mg/day to 20–30 mg/kg/day	Levetiracetam: 10 PBO: 10 Age: 7.62 years (intervention group), 9.82 years (control group) Gender: 17 boys, three girls IQ: 75.75 ± 33.4 (range 29–107) FU: 10 weeks	CGI-AD, ABC, Parent C-YBOCS, CPRS	No significant inter-group difference according to any of the outcome measures, except for teacher ratings on ABC-I (*P*: 0.003). The ABC-I score in the placebo group was significantly reduced at FU (61.59 to 58.94) compared with the intervention group (56.41 to 59.96). Jadad = 4	Small sample size and short study duration
Topiramate						
Rezaei et al (2010)^31^	Parallel-design double-blind placebo-controlled add-on, DSM IV-TR, ADI-R, and ABC-I score ≥12.	Topiramate (100 mg/day to 200 mg/day) + risperidone (0.5–2 mg/day for children up to 40 kg and 3 mg/day for children over 40 kg.	Risperidone + topiramate: 20 Risperidone + PBO: 20 Age: 8.17 years (intervention), 7.85 years (control) Gender: 27 boys, 13 girls IQ: Not specified FU: 8 weeks	ABC-C subscales, ESRS	Of the five subscales of ABC-C, there were significant improvements in the treatment group compared with the control group in three, namely irritability, hyperactivity/ noncompliance and stereotypy (all *P* < 0.05) but not in lethargy/social withdrawal and inappropriate speech, which showed non-significant inter-group difference. Jadad = 5	Small sample size and short study duration. Also, contradictory findings according to the different ABC-C subdomain scores
Valproate						
Hellings et al (2005)^32^	Parallel-design double-blind placebo-controlled, DSM-IV, ADI-R, ADOS	Valproate liquid (250 mg/day to 20 mg/kg/day); mean trough serum level was 77.8 μg/ml at trial end-point.	Valproate: 16 (13 completed) PBO: 14 (12 completed) Age: 11.2 ± 4.2 years (6–20 years) Gender: 20 males, 10 females IQ: 54 (20–137) (24 with IDD, two with borderline IQ, and one each with average and above average IQ, two missing IQ data) FU: 8 weeks	ABC-I, OAS, CGI-I, CGI-S	No significant inter-group difference according to any of the outcome measure scores. The same result was found for children with IDD. Jadad = 4	Small sample size, short study duration, heterogeneous sample and large placebo effect

ABC-C, Aberrant Behaviour Checklist-Community version; ADI-R, Autism Diagnostic Interview-Revised; ADOS, Autism Diagnostic Observation Schedule; ASD, Autistic Spectrum Disorder; AUBC, Autism Behaviour Checklist; CARS, Childhood Autism Rating Scale; CCS, communication composite score; CGI, Clinical Global Impressions Scale; CGI-AD, CGI Scale for Autistic Disorder; CGI-I, CGI Scale - Improvement; CGI-S, CGI-Severity, CPRS, Conners’ Parents Rating Scale; CVP, cognitive verbal/preverbal; C-YBOCS, Children's Yale Brown Obsessive Compulsive Scale; DSM-IV-TR, DSM-IV Text Revision; EL, expressive language; ESRS, Extrapyramidal Symptom Rating Scale; FU, follow-up; IDD; intellectual developmental disabilities; IQ, Intelligence Quotient; OAS-M, Overt Aggression Scale-Modified; PBO, placebo; PL-ADOS, Pre-linguistic Autism Diagnostic Observation Schedule; PEP-3, Psychoeducational Profile third edition; RL, receptive language; VABS, Vineland Adaptive Behavior Scales; YBOCS, Yale-Brown Obsessive Compulsive Scale; YMRS, Young Mania Rating scale.

### Meta-analysis

RevMan version 5.3 software for Windows 10^28^ was used to conduct the meta-analysis. Meta-analysis was carried out for RCTs that had the same outcome measures. Funnel plots and Egger's test^29^ were used to assess publication bias (Supplementary Appendix 5).

### Confidence in the cumulative estimate

The quality of evidence was assessed across the risk-of-bias domains of consistency, directness, precision and publication bias. Any studies that were deemed to be of low quality were excluded from the review. A Measurement Tool to Assess Systematic Reviews 2 (AMSTAR 2, Supplementary Appendix 6)^30^ was used to assess the overall quality of the systematic review and meta-analysis. No ethical approval was necessary for this review.

## Results

### Search findings

**Fig. 1:**
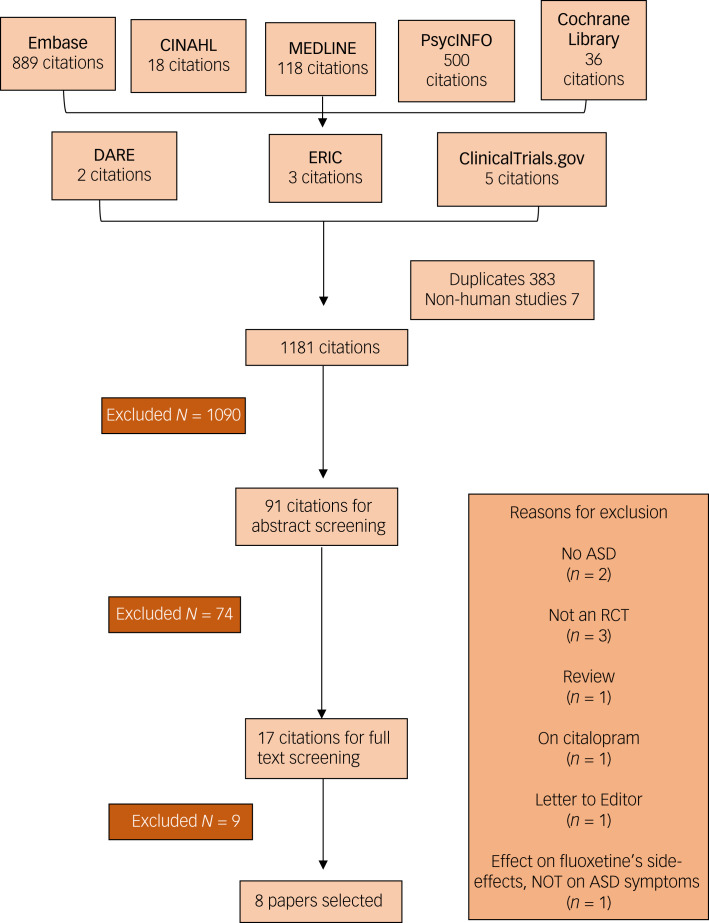
PRISMA flow chart of the article selection process.

The literature search produced 1571 results, from which 383 duplicates and 7 non-human studies were removed. A total of 1181 titles were screened, and 91 abstracts were selected for further screening. In the abstract screening, 74 articles were excluded and only 17 articles underwent full-text screening. Nine further articles were removed; reasons for removal can be found in Fig. 1. Eight papers were selected for the review. Three articles were on divalproex sodium;^24,25,27^ one each on lamotrigine,^23^ topiramate,^31^ and sodium valproate^32^ respectively; and two on levetiracetam.^26,33^

### Description of the study population

The studies included 310 participants in total, with 209 male and 62 female participants. One study did not specify the number of males and females in their study.^24^ Almost all (*n* = 6) RCTs included children and adolescents only (age range from 3 to 18 years); two RCTs included children, adolescents and adults^24,32^ (age range from 20–40 years). Participants with IDD were included in four RCTs,^24–26,32^ but IQ data were not presented in the other four.^23,27,31,33^ Only one study^32^ recruited predominantly children with IDD (24 of the 30 participants) and reported data on the IDD participants separately. Most studies were parallel-design placebo-controlled double-blind RCTs, except for one which was single-blind and not placebo-controlled.^33^ One study was a non-inferiority study in which divalproex sodium was compared with risperidone rather than placebo,^27^ and one was an add-on study where either topiramate or placebo was added to risperidone.^31^ Five studies (63%)^23–26,32^ were from the USA and there was one each from China,^33^ Ukraine^27^ and Iran,^31^ respectively. Of the eight studies, four (50%) were part-funded by pharmaceutical companies;^23,24,26,32^ however, the source of funding was not mentioned in two studies (25%) and, therefore, sponsorship by pharmaceutical companies could not be ruled out.^27,33^ The remaining two studies (25%)^25,31^ were not funded by pharmaceutical companies.

### Criteria used for diagnosing ASD

Different studies used different methods to diagnose ASD. The DSM-IV was used in four studies, two involving divalproex sodium,^24,27^ and one each for levetiracetam^26^ and sodium valproate.^32^ The DSM-IV text revision (DSM-IV-TR) was used in two studies, involving divalproex sodium^25^ and topiramate,^31^ respectively. DSM-V criteria were used in one study involving levetiracetam.^33^ Furthermore, seven studies used the Autism Diagnostic Interview-Revised (ADI-R) (divalproex sodium,^24,25,27^ lamotrigine,^23^ levetiracetam,^26^ topiramate^31^ and sodium valproate^32^) and five used the Autism Diagnostic Observation Schedule (ADOS) (divalproex sodium,^24,25,27^ levetiracetam^26^ and sodium valproate^32^) to confirm the diagnosis of ASD.

### Data on IDD

Four^24–26,32^ studies recruited people with IDD, but only one study on valproate provided separate data on participants with IDD (Table 1).^32^ Mean IQ values and ranges can be found in Table 1. IQ was measured by the Leiter international performance scale-revised,^25,26^ Wechsler Intelligence Scale for Children,^26,32^ Stanford Binet test^32^ or Vineland Adaptive Behavior Scales (VABS).^32^ The type of scale used to assess IQ was not mentioned in one study.^24^ IQ was not specified in four studies,^23,27,31,33^ although two studies suggested that participants with IDD could be included if a diagnosis of ASD could be confirmed.^23,31^

### Outcome measures used in the included studies

A variety of outcome measures were used in the included studies. The most common outcome measures used were the Aberrant Behaviour Checklist (ABC)/ABC-irritability (ABC-I),^34^ followed by the CGI-I,^35^ and Children-Yale-Brown Obsessive Compulsive Scale (C-YBOCS)^36^ and OAS/OAS-M.^37,38^ ABC was used in five RCTs.^23,25–27,31^ CGI-I was used in four RCTs,^25–27,32^ while C-YBOCS^24–26^ and OAS-M were used in three RCTs,^25,27,32^ respectively. Other outcome measures used are shown in Table 1. It is beyond the scope of this paper to go into the details of the psychometric properties of the outcome measures, but some of these have been reviewed by Budimirovic and colleagues.^39^

### A narrative synthesis of the data

#### Divalproex sodium

All three RCTs on divalproex sodium used a double-blind parallel design. Two studies^24,25^ compared divalproex sodium with placebo, and one^27^ compared it with risperidone. In the first placebo-controlled study of 12 children and one adult (age 40 years), divalproex sodium was significantly better than placebo in improving repetitive behaviours as measured by C-YBOCS.^24^ In the second placebo-controlled trial by the same group on 27 children, a marginally statistically significant improvement was seen with divalproex sodium compared with placebo in reducing irritability when measured by the ABC scale, but no such significant reduction in irritability (we are, however, not aware of any irritability measure in OAS-M) was found when measured by OAS-M.^25^ This finding did not change when IQ difference was controlled for. The responder rate by CGI-I for irritability (62.5%) and general core autism symptoms domains (CGI-AU; 12.5%) were higher in the divalproex sodium group, but only the former was significant. Responders had higher valproate blood levels compared with non-responders. There were also no significant inter-group differences between divalproex sodium and placebo groups on daily functioning as measured by VABS^40^ and on repetitive behaviours as measured by C-YBOCS.^25^ By contrast, the third study on divalproex sodium on 86 children found that risperidone was significantly better than divalproex sodium in reducing irritability, stereotypy, hyperactivity and non-compliant behaviours.^27^ Risperidone was also significantly better than divalproex sodium in improving irritability and aggression as measured by OAS-M and improving ASD symptoms as measured by CGI-I. However, the Jadad score for this study was poor.

#### Lamotrigine

Only one study on 28 children compared lamotrigine with placebo and found no significant inter-group differences between the placebo and lamotrigine groups on ASD core symptoms and associated behaviours as measured by the ABC, Childhood Autism Rating Scale (CARS),^41^ Autism Behaviour Checklist (AUBC),^42^ and Pre-Linguistic ADOS (PL-ADOS).^43^ Lamotrigine, however, was marginally better than placebo at improving adaptive social behaviours as measured by VABS.^23^

#### Levetiracetam

Two RCTs investigated the efficacy of levetiracetam. One study was a 6 month physician-blinded RCT which compared a combination of levetiracetam and PEP with PEP alone,^33^ whereas the other was a 10 week double-blind RCT which compared levetiracetam with placebo.^26^ In the 6 month RCT trial on 67 children, the combination of levetiracetam and PEP showed significantly better improvements in ASD core symptoms as measured by CARS and AUBC, and other behaviours including expressive language, receptive language and cognitive verbal/preverbal function as measured by the Psychoeducational Profile-third edition (PEP-3 Chinese version).^44^ The combination was also significantly better than PEP alone at controlling subclinical epileptiform discharge, which was present at baseline for all participants but absent during follow-up in 24 of the 32 participants (75%) in the intervention group compared with five of the 35 participants (14.3%) in the control group.^33^ In the other 10 week RCT trial of 20 children, no significant difference was found between levetiracetam and placebo in improving associated behaviours, particularly irritability, lethargy, stereotypy, hyperactivity and inappropriate speech according to parent-rated ABC scores. However, on teacher ratings, only the irritability subscale showed a significant difference, with the placebo significantly better than levetiracetam at improving irritability. No inter-group significant difference was seen for repetitive behaviours as measured by C-YBOCS or change in CGI-I.^26^

#### Topiramate

Only one RCT^31^ was included that compared topiramate add-on versus placebo add-on to risperidone in 40 children. Risperidone was titrated up to 2 mg/day for children weighing less than 40 kg and 3 mg/day for children weighing above 40 kg. The combination of topiramate and risperidone was significantly better than the combination of risperidone and placebo at reducing irritability, stereotypy and hyperactivity/noncompliant behaviours, but no significant inter-group differences were observed for inappropriate speech and lethargy/social withdrawal.

#### Sodium valproate

Only one RCT was included that compared sodium valproate with placebo in an 8 week parallel-design double-blind placebo-controlled trial on 30 children, adolescents and adults (up to age 20 years). No significant inter-group difference was reported in irritability as measured by ABC, aggressive behaviours as measured by OAS, or CGI-I or CGI-severity.^32^

#### Adverse events

Various adverse events were reported in different studies (Table 2), but most of these (63%) were mild to moderate in all studies. There were no significant inter-group differences in the rate of adverse events, except for one study on topiramate^31^ and one study on sodium valproate.^32^ In one of the divalproex sodium trials,^25^ one person had clinically significant weight gain in the intervention group, but overall there was no significant inter-group difference in the rate of weight gain. In another study on levetiracetam,^33^ in the treatment group one child each reported irritability, fatigue and somnolence, and anorexia, respectively, which resolved after dose reduction or spontaneously. Only two participants dropped out owing to adverse events; one was in the divalproex sodium trial^25^ and the other was in the sodium valproate trial^32^ (Table 2). There may have been an added problem of participants’ reporting adverse events as most of them were children.

**Table 2:** Adverse events reported in the included studies

Study author and date of publication	Any statistically significant inter-group differences in type or number of adverse events reported	Any drop-out due to adverse events	Reported adverse events in the study
Divalproex sodium			
Hollander et al (2006)^24^	No statistically significant inter-group difference in the numbers or types of adverse events.	None	Irritability, weight gain, anxiety and aggression reported in both groups.
Hollander et al (2010)^25^	No statistically significant inter-group difference in the numbers or types of adverse events.	One participant dropped out of the study owing to paradoxical increase in irritability associated with insomnia	Divalproex sodium group: insomnia, weight gain, headache, rash, polyuria, agitation and infections. Placebo group: insomnia, weight gain, agitation, hypersomnolence and infection.
Martsenkovsky (2014)^27^	No statistically significant inter-group difference in the numbers or types of adverse events.	None	Divalproex sodium group: weight gain, autonomic symptoms and sedation. Risperidone group: weight gain, autonomic symptoms, sedation, extrapyramidal symptoms and symptoms related to hyperprolactinemia.
Lamotrigine			
Belsito et al (2001)^23^	No statistically significant inter-group difference in the numbers or types of adverse events.	None	Insomnia, rash and hyperactivity reported in both groups.
Levetiracetam			
Wang et al (2017)^33^	Only the treatment group (levetiracetam + psychoeducation) was assessed for adverse effects.	None	Anorexia, irritability, fatigue and somnolence.
Wasserman et al (2006)^26^	No statistically significant inter-group difference in the numbers or types of adverse events.	None	Levetiracetam group: agitation, aggression, hyperactivity, impulsivity, loss of appetite, self-injurious behaviour, weight gain, weight loss. Placebo: agitation, aggression, enuresis and insomnia.
Topiramate			
Rezaei et al (2010)^31^	A significantly higher rate of somnolence (35% *v*. 5%) and decreased appetite (35% *v*. 5%) in the topiramate + risperidone group compared with the topiramate-alone group.	None	Somnolence, decreased appetite, increased appetite, paraesthesia, dizziness, insomnia, nausea and sedation reported in both groups.
Sodium valproate			
Hellings et al (2005)^32^	The only adverse event reaching significant level was increased appetite (*P* = 0.03); 56% in the valproate group *v*. 14% in the control group.	One participant dropped out of the study owing to a spreading skin rash on the trunk and extremities, which resolved after discontinuation of sodium valproate.	Valproate group: gastrointestinal complaints of nausea, vomiting, abdominal discomfort, constipation and diarrhoea, and other complaints including drowsiness, lethargy, headache, chills, increased appetite, weight gain, skin rash and fever. Elevation of ammonia above the normal range was observed, and one participant's parent reported cognitive slowing and slurred speech at times. Placebo group: gastrointestinal complaints of nausea, vomiting, abdominal discomfort, constipation and diarrhoea, and other complaints including drowsiness, headache, chills, increased appetite, weight gain, skin rash and fever.

### Quality of the included papers

**Fig. 2:**
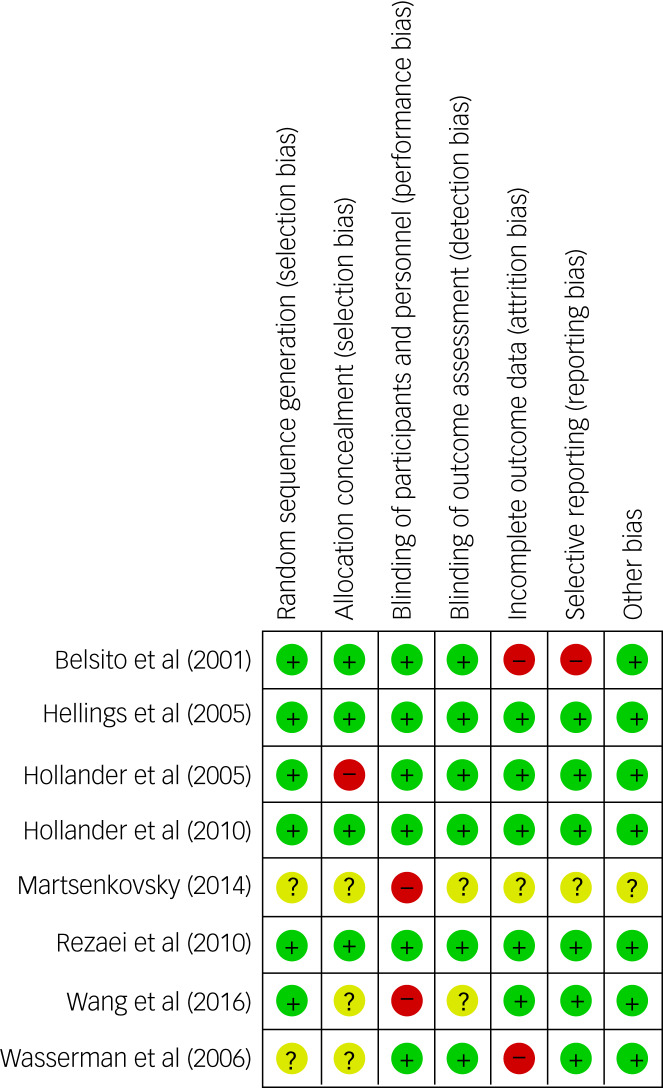
Cochrane risk-of-bias summary scores and graph.

The Cochrane risk-of-bias analysis showed that five^23,24,26,27,33^ out of eight studies (63%) had a high risk for at least one item, of which one (12.5%) showed high risk for two items (Fig. 2). Six of the eight studies (75%) received a Jadad score of less than 5, with Jadad scores ranging from 0 to 5 and a mean of 3.75.^24–27,32,33^ Although the funnel plot of ABC-I appeared to be asymmetrical (Supplementary Appendix 3), the Egger's test of publication bias was not significant, suggesting no publication bias (*P* = 0.95). Eggers’ test for publication bias could not be conducted for the OAS/OAS-M and CGI-I meta-analyses, as the meta-analyses included only two studies each. The funnel plots for the OAS/OAS-M and CGI-I meta-analyses were also difficult to interpret as only two studies were included. Therefore, large-scale RCTs on the use of mood stabilisers in ASD are needed in the future. The overall quality of the systematic review and the meta-analysis was high based on the AMSTAR 2 scale, showing only one non-critical weakness (Supplementary Appendix 6).

### Meta-analysis

**Fig. 3:**
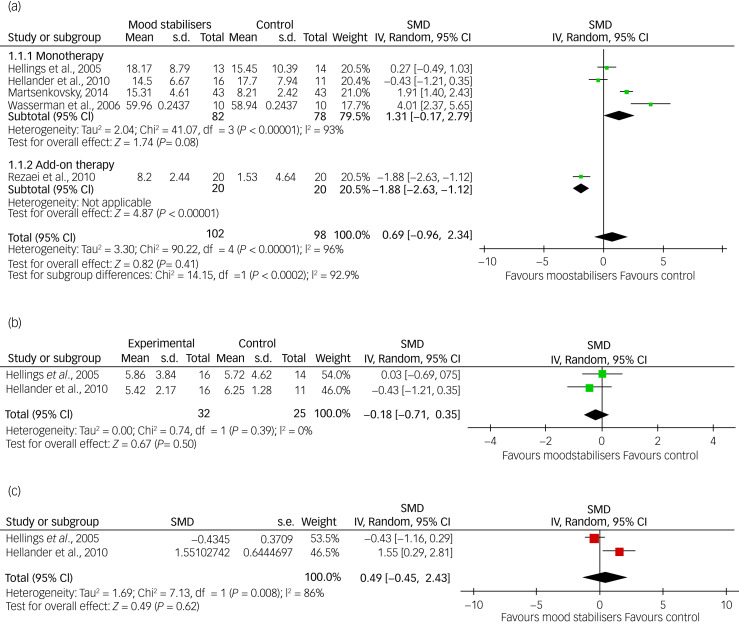
Forest plots (a) ABC-I meta-analysis, (b) OAS/OAS-M meta-analysis and (c) CGI-I.

Three meta-analyses were possible with the available data based on ABC-I, CGI-I and OAS/OAS-M scores, respectively. Data on irritability (ABC-I) were available from five of the eight RCTs (62.5%): two on divalproex sodium,^25,27^ and one each on topiramate,^31^ sodium valproate^32^ and levetiracetam,^26^ respectively. However, as the topiramate study was an add-on study and the rest were on a single medication, the data from the former study were analysed separately in a subgroup analysis (Fig. 3). For CGI-I and OAS/OAS-M, data were available from two studies (25%): one on sodium valproate^32^ and one on divalproex sodium^25^ for each outcome, respectively.

The meta-analysis of pooled ABC-I scores showed that there was no significant inter-group difference between mood stabilisers and placebo (SMD = 0.70, 95% CI = −0.93 to 2.33, *P* = 0.40). Only one study that used topiramate as an add-on therapy with risperidone was significant, whereas four studies comparing mood stabiliser monotherapy with placebo were not significant. However, heterogeneity was high (*I^2^* = 96%). A sensitivity analysis and removal of studies with a high risk of bias did not show any significant inter-group difference. The pooled data showed no statistically significant inter-group difference for either the OAS/OAS-M (SMD = −0.18, 95% CI = −0.71 to 0.35, *P* = 0.50) or the CGI-I (SMD = 0.49, 95% CI = −1.45 to 2.43, *P* = 0.62); although the heterogeneity was low for OAS/OAS-M at *I^2^* = 0%, it was high for CGI-I at *I^2^* = 86% (Fig. 3).

## Discussion

Our systematic review included eight RCTs on mood stabilisers involving 310 participants with ASD of any age (3–40 years), including three on divalproex sodium, two on levetiracetam, and one each on lamotrigine, sodium valproate and topiramate (as an add-on to risperidone). This compares with Hirota and colleagues’ systematic review that included seven RCTs on anti-epileptic medications involving 117 children and adolescents, including two studies on divalproex sodium; one each on sodium valproate, lamotrigine and levetiracetam; and one each combining topiramate and risperidone, and sodium valproate and fluoxetine.

### Study design

Of the three studies included in our review on divalproex sodium, two were from the same group^24,25^ that used a placebo-controlled parallel-design RCT, but the third was a non-inferiority comparison between divalproex and risperidone rather than placebo.^27^ The two studies on levetiracetam in our review both used a parallel design, but one was placebo-controlled and double-blind,^26^ and the other was a single-blind (physician-blind) comparison of combined levetiracetam and PEP with PEP alone.^33^ Single studies on lamotrigine^23^ and sodium valproate^32^ used a parallel-design placebo-controlled RCT, whereas the single study on topiramate was a comparison between two groups, both of which received risperidone, one with added topiramate and the other with added placebo.^31^

### Outcomes measured

Although our search terms included bipolar and other psychiatric disorders, and the review was on mood stabilisers, surprisingly we did not find any RCT on bipolar or any other psychiatric disorder. The included studies reported the effect of mood stabilisers primarily on ASD core symptoms such as repetitiveness as measured by CYBOCS^36^ and language impairment and other associated behaviours such as irritability and aggression as measured by ABC-C and OAS/OAS-M.^34,37,38^

### Characteristics of the study population

Although our search terms included studies on people with ASD of all ages, the search generated primarily studies involving children, except one study^32^ which included participants between 6 and 20 years and another^24^ which included one adult (age 40 years) and 12 children.^24^ Some studies mentioned the IQ range of participants, but others did not. Even in those studies that included participants with IDD, no separate data were presented on participants with IDD, except in one study^32^ which included predominantly children with IDD. Most studies were from the USA and were part-funded by pharmaceutical companies, although the influence of pharmaceutical companies on the findings of these studies is not known.

### Overall findings

Two studies on divalproex versus placebo showed a significantly better outcome in the treatment group compared with the placebo group on most outcome measures but not all.^24,25^ However, the third study found a significantly better outcome in the risperidone group compared with the divalproex group.^27^ There is some preliminary evidence to suggest that risperidone and to some extent aripiprazole may be effective in treating associated behaviours such as irritability and aggression in children with ASD.^45–47^ Of the two studies on levetiracetam included in our review, the combination of levetiracetam and PEP resulted in a significantly better outcome when compared with PEP alone, including improvements in cognition and also in subclinical electroencephalogram (EEG) abnormalities which were present at baseline in all participants.^33^

Apart from one study^25^ with a very small number of participants with EEG abnormalities, no study in our review reported the effect of the intervention on subclinical EEG abnormalities. However, the second study on levetiracetam showed no significant inter-group difference.^26^

The only placebo-controlled double-blind RCT included in our review on lamotrigine^23^ showed a marginally significant difference in improvement in adaptive social behaviours but did not show any inter-group difference for any of the other outcomes. The only placebo-controlled double-blind RCT included in our review on sodium valproate^32^ did not show any significant inter-group difference in either irritability or aggression as measured by OAS and ABC-I.

The only placebo-controlled double-blind add-on RCT included in our review on topiramate^31^ found equivocal results. The combination of risperidone and topiramate was significantly better than the combination of risperidone and placebo in reducing irritability, stereotypy and hyperactivity/noncompliance behaviours, but no inter-group difference was observed for inappropriate speech and lethargy/social withdrawal.

### Conclusions from the overall findings

Overall, one study on levetiracetam^33^ showed a positive result when compared with PEP alone, one study on lamotrigine^23^ showed a marginal positive result (*P* = 0.045) when compared with placebo, and two studies on divalproex versus placebo by the same group^24,25^ showed a positive result. Although one of the studies on divalproex versus placebo showed a significant result according to the primary outcome measure, it failed to show any significant inter-group difference according to four secondary outcome measures.^25^ This was also the case for the lamotrigine study, which failed to show any significant inter-group difference according to five other outcome measures.^23^ The sample sizes were also very small in the two studies on divalproex versus placebo (13 and 27, respectively). Single placebo-controlled studies on valproate,^32^ and levetiracetam^26^ each showed negative results, and one comparison study of divalproex sodium with risperidone showed a negative finding for divalproex.^27^ The only study on topiramate showed an equivocal result.^31^

### Meta-analysis findings

It was possible to pool data on ABC-I scores from four placebo-controlled monotherapy studies on divalproex sodium (*n* = 2),^24,27^ sodium valproate (*n* = 1)^32^ and levetiracetam (*n* = 1)^26^ and one combination study with topiramate and risperidone^31^ for a meta-analysis. Similarly, data could be pooled for meta-analysis from two studies each using OAS/OAS-M and CGI-I data, respectively.^25,32^ None of the meta-analyses showed any statistically significant inter-group difference. Although many different adverse events were reported in the included studies in both the intervention and the control groups, only two studies^31,32^ showed a significant inter-group difference.

### Comparison with the previous systematic review

Hirota and colleagues searched for studies on anti-epileptic medication only compared with placebo, whereas we searched for studies on all mood stabilisers, including anti-epileptic medications, compared with any other intervention including placebo. Hirota and colleagues’ review included studies on a total of 171 participants, whereas our review included 310 people with ASD of all ages. Also, whereas Hirota and colleagues included seven anti-epileptic medications, we included eight mood stabilisers, although all of them were anti-epileptic medications as we did not find any study on lithium. We excluded one study that was included in Hirota and colleagues’ review,^48^ as this one was on the effect of levetiracetam as an add-on therapy on the adverse events associated with fluoxetine. We included six studies that were included in Hirota and colleagues’ study.^23–26,31,32^

We added two studies that were not included in Hirota and colleagues’ study.^27^^,^^33^ The first one compared the combined effects of levetiracetam and PEP with PEP alone.^33^ This study found improvement in core ASD symptoms at 6 month follow-up in both groups, but the combined levetiracetam and PEP group showed a significantly better outcome compared with the PEP-alone group. The second study^27^ compared the efficacy of divalproex sodium with risperidone instead of placebo and found that risperidone was significantly better than divalproex sodium in improving both ASD core symptoms and associated behaviours such as irritability and stereotypy. The overall findings of Hirota and colleagues and our review were the same in that no definitive evidence of the superiority of mood stabilisers over placebo could be established. However, these findings have to be interpreted within the context of several methodological limitations.

### Methodological flaws in the included studies

The sample sizes in individual studies included in this review were small (only two studies included more than 50 participants, namely 66 and 86, respectively);^27,33^ thus, there was a lack of power to detect small effect sizes. Meta-analyses to some extent address this problem of sample size, but given the variety of outcome measures used in different studies, it was not possible to pool data from all eight included studies. Therefore, the total number of participants included in the meta-analysis (*n* = 57–203 if the add-on study was included) may still not have provided adequate power to detect a small effect size. However, very small effect sizes may not be clinically significant, as one has to weigh the adverse effects that will affect the person's quality of life against improvement in behaviour.

Also, two of the meta-analyses in our study showed high heterogeneity, and a sensitivity analysis did not change these findings. Apart from the two small placebo-controlled studies carried out by the same group on divalproex sodium, no study showed any significant inter-group difference.

Another problem was the short follow-up period used in the included studies, which may not have allowed enough time to show an effect of the intervention. Although one study of a combination of levetiracetam and PEP followed up participants at 6 months, other studies used a much shorter follow-up period (8–18 weeks). However, the former study was not placebo-controlled or double-blind, and in the same study both groups showed improvement at follow-up.

There was also a problem with the dosages and the blood levels of the medications used for dose titration in the included studies; these may not have been adequate to detect a small effect size. However, a balance has to be struck between efficacy and adverse effects in determining the correct dose.

The use of different outcome measures also made interpretation of findings difficult; in particular, in some studies, different scales used to measure the same symptoms gave contradictory results.^25^ Similarly, in some studies, scoring by parents and teachers on the same scale showed contradictory results.^26^

One has to also consider the potential of strong placebo effects on most of the outcome measures, as shown in Luu and colleagues’ review of RCTs on one neurodevelopmental disorder.^49^

### Strengths

Our systematic review used a very stringent methodology, including hand-searching of 12 relevant journals, and should have captured the most relevant RCTs. This is reflected in the full score in AMSTAR2 apart from one noncritical item for not including grey literature. We excluded grey literature as it was not possible to assess its quality using the Cochrane Risk of Bias instrument. It is still possible that relevant papers may have been missed; in addition, only English-language papers were included. Another problem is that studies with positive findings tend to find their way to publication more often than those with negative findings, creating a publication bias. However, Egger's test in our review did not show publication bias. Another strength of this review is that it was registered on PROSPERO so the study protocol can be accessed by anyone (www.crd.york.ac.uk/prospero).

### Limitations

Several drawbacks have to be considered while interpreting the data of this systematic review. Different studies used different outcome measures, which produced heterogeneity when the findings were combined. As a result, we could only pool data for meta-analysis from those studies that used the same outcome measure, for instance, ABC-I, OAS/OAS-M, and CGI-I were reported in only five (63%) of the eight RCTs. Although the meta-analysis of the OAS/OAS-M forest plot showed no heterogeneity, the forest plot involving data from ABC-I and CGI-I scores showed high heterogeneity (86–96%). Another major problem was the small sample sizes in most studies. For example, no study included more than 100 participants, and only two (25%) recruited more than 50 participants. This made the findings from most of these studies difficult to generalise. The quality of individual studies was poor. The Cochrane risk-of-bias analysis showed that five studies (63%) scored as high risk for at least one item, of which one (13%) showed high risk for two items. Six studies (75%) received a Jadad score of less than five, making the interpretation of findings difficult. Also, a number of studies were part-funded by pharmaceutical companies, although their influence on the study findings is unknown.

## Data Availability

Data availability does not apply to this article as no new data were created or analysed in this study.

## Supplementary material

For supplementary material accompanying this paper visit http://doi.org/10.1192/bjo.2022.18. click here to view supplementary material

## References

[1] American Psychiatric Association. Diagnostic and Statistical Manual of Mental Disorders: DSM-5. American Psychiatric Publishing, 2013.

[2] World Health Organization. International Classification of Diseases for Mortality and Morbidity Statistics 11th Revision. WHO, 2019. Available from: https://icd.who.int/browse11/l-m/en [cited 11 Mar 2021].

[3] Simonoff E, Pickles A, Charman T, Chandler S, Loucas T, Baird G. Psychiatric disorders in children with autism spectrum disorders: prevalence, comorbidity, and associated factors in a population-derived sample. J Am Acad Child Adolesc Psychiatry 2008; 47: 921–9. 18645422 10.1097/CHI.0b013e318179964f

[4] Lai MC, Kassee C, Besney R, Bonato S, Hull L, Mandy W, Prevalence of co-occurring mental health diagnoses in the autism population: a systematic review and meta-analysis. Lancet Psychiatry 2019; 6: 819–29. 31447415 10.1016/S2215-0366(19)30289-5

[5] Lugo-Marína J, Magán-Magantob M, Rivero-Santanac A, Cuellar-Pompac L, Alviania M, Jenaro-Riob C, Prevalence of psychiatric disorders in adults with autism spectrum disorder: a systematic review and meta-analysis. Res Autism Spectr Disord 2019; 59: 22–33.

[6] Coury DL, Anagnostou E, Manning-Courtney P, Reynolds A, Cole L, McCoy R, Use of psychotropic medication in children and adolescents with autism spectrum disorders. Pediatrics 2012; 130(suppl 2): s69–76. 23118256 10.1542/peds.2012-0900D

[7] Bachmann CJ, Manthey T, Kamp-Becker I, Glaeske G, Hoffmann F. Psychopharmacological treatment in children and adolescents with autism spectrum disorders in Germany. Res Dev Disabil 2013; 34: 2551–63. 23747941 10.1016/j.ridd.2013.05.028

[8] Murray ML, Hsia Y, Glaser K, Simonoff E, Murphy DG, Asherson PJ, Pharmacological treatments prescribed to people with autism spectrum disorder (ASD) in primary health care. Psychopharmacology 2014; 231: 1011–21. 23681164 10.1007/s00213-013-3140-7 PMC3932167

[9] Jobski K, Höfer J, Hoffmann F, Bachmann C. Use of psychotropic drugs in patients with autism spectrum disorders: a systematic review. Acta Psychiatr Scand 2017; 135: 8–28. 27624381 10.1111/acps.12644

[10] Deb S, Roy M, Lee R, Majid M, Limbu B, Santambrogio J, Randomised controlled trials of antidepressant and anti-anxiety medications for people with autism spectrum disorder: systematic review and meta-analysis. BJPsych Open 2021; 7: e179. 1–15. 34593083 10.1192/bjo.2021.1003 PMC8503912

[11] Huband N, Ferriter M, Nathan R, Jones H. Antiepileptics for aggression and associated impulsivity. Cochrane Database Syst Rev 2010; 2: CD003499. 10.1002/14651858.CD003499.pub3 PMC4163499 20166067

[12] Jones RM, Arlidge J, Gillham R, Reagu S, van den Bree M, Taylor PJ. Efficacy of mood stabilisers in the treatment of impulsive or repetitive aggression: systematic review and meta-analysis. Br J Psychiatry 2011; 198: 93–8. 21282779 10.1192/bjp.bp.110.083030

[13] Deb S, Deb T. Neuropsychiatry of aggression. In Oxford Textbook of Neuropsychiatry (eds R Faruqui, M Bodani, N Agrawal): 379–91. Oxford University Press, 2020.

[14] Dhindsa A. Valproate in dementia: time to move on? BJPsych Adv 2019; 25: 145–9.

[15] National Institute for Health and Care Excellence. Dementia: Assessment, Management and Support for People Living with Dementia and Their Carers (NG97). NICE, 2018. 30011160

[16] Hirota T, Veenstra-VanderWeele J, Hollander E, Kishi T. Antiepileptic medications in autism spectrum disorder: a systematic review and meta-analysis. J Autism Dev Disord 2014; 44: 948–57. 24077782 10.1007/s10803-013-1952-2

[17] Lefebvre C, Glanville J, Briscoe S, Littlewood A, Marshall C, Metzendorf M-I, Chapter 4: searching for and selecting studies. In Cochrane Handbook for Systematic Reviews of Interventions Version 6.0 (Updated July 2019) (eds JPT Higgins, J Thomas, J Chandler, M Cumpston, T Li, MJ Page): 67–108. John Wiley & Sons, 2019.

[18] Li T, Higgins JPT, Deeks JJ. Chapter 5: Collecting data. In Cochrane Handbook for Systematic Reviews of Interventions Version 6.0 (Updated July 2019) (eds JPT Higgins, J Thomas, J Chandler, M Cumpston, T Li, MJ Page, VA Welch): 109–42. John Wiley & Sons, 2019.

[19] Mendeley [Computer program]. Mendeley Desktop for Windows. Mendeley, 2008. Available from: https://www.mendeley.com/download-desktop-new/.

[20] Higgins JPT, Savović J, Page MJ, Elbers RG, Sterne JAC. Chapter 8: assessing risk of bias in a randomized trial. In Cochrane Handbook for Systematic Reviews of Interventions Version 6.1 (Updated September 2020) (eds JPT Higgins, J Thomas, J Chandler, M Cumpston, T Li, MJ Page, VA Welch): 205–28. John Wiley & Sons, 2020.

[21] Jadad AR, Moore RA, Carroll D, Reynolds DJ, Gavaghan DJ, McQuay HJ. Assessing the quality of reports of randomised clinical trials: is blinding necessary? Controlled Clin Trials 1996; 17: 1–12. 8721797 10.1016/0197-2456(95)00134-4

[22] Deeks JJ, Higgins JPT, Altman DG. Chapter 10: analysing data and undertaking meta-analyses. In Cochrane Handbook for Systematic Reviews of Interventions Version 6.0 (Updated July 2019) (eds JPT Higgins, J Thomas, J Chandler): 241–84. John Wiley & Sons, 2019.

[23] Belsito KM, Law PA, Kirk KS, Landa RJ, Zimmerman AW. Lamotrigine therapy for autistic disorder: a randomized, double-blind, placebo-controlled trial. J Autism Dev Disord 2001; 31: 175–81. 11450816 10.1023/a:1010799115457

[24] Hollander E, Soorya L, Wasserman S, Esposito K, Chaplin W, Anagnostou E. Divalproex sodium vs. placebo in the treatment of repetitive behaviours in autism spectrum disorder. Int J Neuropsychopharmacol 2006; 9: 209–13. 16316486 10.1017/S1461145705005791

[25] Hollander E, Chaplin W, Soorya L, Wasserman S, Novotny S, Rusoff J, Divalproex sodium vs placebo for the treatment of irritability in children and adolescents with autism spectrum disorders. Neuropsychopharmacol 2010; 35: 990–8. 10.1038/npp.2009.202 PMC2846602 20010551

[26] Wasserman S, Iyengar R, Chaplin WF, Watner D, Waldoks SE, Anagnostou E, Levetiracetam versus placebo in childhood and adolescent autism: a double-blind placebo-controlled study. J Clin Psychopharmacol 2006; 21: 363–7. 10.1097/01.yic.0000224787.13782.0f 17012983

[27] Martsenkovsky I. Divalproex sodium and risperidone in the treatment of cognitive, behavioral and social dysfunction in preschool children with PDD and ADHD. Eur Neuropsychopharmacol 2014; 24: S721–2.

[28] Review Manager (RevMan) [Computer program]. Version 5.3. Copenhagen: The Nordic Cochrane Centre, The Cochrane Collaboration. 2014.

[29] Egger M, Smith GD, Schneider M, Minder C. Bias in meta-analysis detected by a simple, graphical test. BMJ 1997; 315: 629–34. 9310563 10.1136/bmj.315.7109.629 PMC2127453

[30] Shea BJ, Reeves BC, Wells G, Thuku M, Hamel C, Moran J, AMSTAR 2: a critical appraisal tool for systematic reviews that include randomised or nonrandomised studies of healthcare interventions, or both. BMJ 2017; 358: j4008. 28935701 10.1136/bmj.j4008 PMC5833365

[31] Rezaei V, Mohammadi MR, Ghanizadeh A, Sahraian A, Tabrizi M, Rezazadeh SA, Double-blind, placebo-controlled trial of risperidone plus topiramate in children with autistic disorder. Prog Neuropsychopharmacol Biol Psychiatry 2010; 34: 1269–72. 20637249 10.1016/j.pnpbp.2010.07.005

[32] Hellings JA, Weckbaugh M, Nickel EJ, Cain SE, Zarcone JR, Reese RM, A double-blind, placebo-controlled study of valproate for aggression in youth with pervasive developmental disorders. J Child Adolesc Psychopharmacol 2005; 15: 682–92. 16190799 10.1089/cap.2005.15.682

[33] Wang M, Jiang L, Tang X. Levetiracetam is associated with decrease in subclinical epileptiform discharges and improved cognitive functions in pediatric patients with autism spectrum disorder. Neuropsychiatr Dis Treat 2017; 13: 2321. 28919764 10.2147/NDT.S143966 PMC5587198

[34] Aman MG, Burrow WH, Wolford PL. The aberrant behavior checklist community: factor validity and effect of subject variables for adults in group homes. Am J Ment Retard 1995; 100: 283–92. 8554775

[35] National Institute of Mental Health. CGI (clinical global impression scale)-NIMH. Psychopharmacol Bull 1985; 21: 839–44.

[36] Goodman WK, Price LH, Rasmussen SA, Mazure C, Fleischmann RL, Hill CL, The Yale-Brown obsessive-compulsive scale: I. development, use, and reliability. Arch Gen Psychiatry 1989; 46: 1006–11. 2684084 10.1001/archpsyc.1989.01810110048007

[37] Yudofsky SC, Silver JM, Jackson W, Endicott J, Williams D. The overt aggression scale for the objective rating of verbal and physical aggression. Am J Psychiatry 1986; 143: 35–9. 3942284 10.1176/ajp.143.1.35

[38] Kay SR, Wolkenfelf F, Murrill LM. Profiles of aggression among psychiatric patients: I. nature and prevalence. J Nerv Ment Dis 1988; 176: 539–46. 3418327 10.1097/00005053-198809000-00007

[39] Budimirovic DB, Berry-Kravis E, Erickson CA, Hall SS, Hessl D, Updated report on tools to measure outcomes of clinical trials in fragile X syndrome. J Neurodevelop Disorders 2017; 9: 14. 10.1186/s11689-017-9193-x PMC5467057 28616097

[40] Sparrow S, Balla D, Cicchetti DV. Vineland Adaptive Behavior Scales (Survey Form). American Guidance Service, 1984.

[41] Schopler E, Reichler R, Renner B. Childhood Autism Rating Scale (CARS). Western Psychological Services, 1988.

[42] Krug DA, Arick J, Almond P. Autism Behavior Checklist Record Form. PRO-ED, Inc, 1993.

[43] DiLavore PC, Lord C, Rutter M. The pre-linguistic autism diagnostic observation schedule. J Autism Dev Disord 1995; 25: 355–79. 7592249 10.1007/BF02179373

[44] Chen KL, Chiang FM, Tseng MH, Fu CP, Hsieh CL. Responsiveness of the psychoeducational profile-third edition for children with autism spectrum disorders. J Autism Dev Disord 2011; 41: 1658–64. 21336523 10.1007/s10803-011-1201-5

[45] Unwin GL, Deb S. Efficacy of atypical antipsychotic medication in the management of behaviour problems in children with intellectual disabilities and borderline intelligence: a systematic review. Res Dev Disabil 2011; 32: 2121–33. 21856116 10.1016/j.ridd.2011.07.031

[46] Deb S, Farmah BK, Arshad E, Deb T, Roy M, Unwin GL. The effectiveness of aripiprazole in the management of problem behaviour in people with intellectual disabilities, developmental disabilities and/or autistic spectrum disorder–a systematic review. Res Dev Disabil 2014; 35: 711–25. 24405794 10.1016/j.ridd.2013.12.004

[47] Ji NY, Findling RL. An update on pharmacotherapy for autism spectrum disorder in children and adolescents. Curr Opin Psychiatry 2015; 28: 91–101. 25602248 10.1097/YCO.0000000000000132

[48] Anagnostou E, Esposito K, Soorya L, Chaplin W, Wasserman S, Hollander E. Divalproex versus placebo for the prevention of irritability associated with fluoxetine treatment in autism spectrum disorder. J Clin Psychopharmacol 2006; 26: 444–6. 16855475 10.1097/01.jcp.0000227703.72117.bc

[49] Luu S, Province H, Berry-Kravis E, Hagerman R, Hessl D, Vaidya D, Response to placebo in fragile X syndrome clinical trials: an initial analysis. Brain Sci 2020; 10: 629. 10.3390/brainsci10090629 PMC7563217 32932789

