# Supplementary material for: Randomised controlled trials of mood stabilisers for people with autism spectrum disorder: systematic review and meta-analysis
Source: BJPsych Open. 2022 Feb 24;8(2):e52. doi: 10.1192/bjo.2022.18 (PMC8935918; doi:10.1192/bjo.2022.18)
Supplement: Supplementary file 1 [file S2056472422000187sup001.docx]

**Appendix 1**: **Search terms**

*Mood stabilisers descriptors:*

mood stabilisers* OR mood stabilizers OR lithium* OR lithium carbonate OR lithium citrate OR sodium valproate OR valproate OR valproic acid* OR divalproex sodium OR depakote OR carbamazepine OR oxcarbazepine OR lamotrigine OR keppra OR gabapentin OR vigabatrin OR tiagabine OR topiramate OR levetiracetam OR anticonvulsant medication OR antiepileptic medication

AND

*ASD descriptors:*

child developmental disorder* OR pervasive developmental disorder* OR autis* OR PDD* OR ASD* OR Kanner* OR Asperger* OR Asperger* syndrome OR autism spectrum disorder OR Rett Syndrome OR childhood schizophrenia OR Fragile X syndrome OR neurodevelopmental disorder* OR NDD*.

AND

*Outcome descriptors:*

anxiety OR anxiety disorder OR depression OR obsessive compulsive disorder OR OCD OR generalised anxiety disorder OR phobia OR agoraphobia OR bipolar disorder OR manic depressive psychosis OR  mania OR hypomania OR autism core symptoms OR ASD core symptoms OR ASD symptoms OR autism symptoms OR social interaction OR communication problems OR agitation OR irritability OR aggression OR behavioural problems OR problem behaviors OR challenging behaviour OR behaviour* that challenge OR behaviour of concern OR maladaptive behaviour OR disruptive behaviour OR disturbed behaviour OR distressed behaviour OR stereotypy OR restricted behaviour OR repetitive patterns of behaviour OR restricted interests OR restrictive activities OR social communication OR repetitive behaviour OR communication* OR inattention OR hyperactivity OR insistence on sameness OR sameness OR sleep problem OR insomnia OR self-injurious behaviour OR self-mutilation OR temper tantrum OR tantrum OR aggression to others OR aggression to property OR sexual aggression OR sexual deviance OR mental state OR global improvement OR quality of life OR CGI.

AND

*RCT descriptors:*

clinical trial* OR randomization* OR randomisation OR research design OR randomized controlled trial OR randomi#ed control* trial* OR RCT OR controlled clinical trial OR double-blind procedure OR random* OR trial* OR control* OR blind* OR crossover OR crossover procedure OR crossover trial* OR volunteer* OR placebo* OR randomly OR control* OR ((singl* or doubl* or trebl* or tripl*) adj3 (blind* or mask*)) OR comparative stud* OR psychopharmacology  AND not (animal OR nonhuman) treatment OR effectiveness evaluation OR treatment outcomes OR follow-up studies OR evaluat* adj3 stud*.

Date Range: 1985 (January) to 2021 (June)

**Appendix 2**: **Eligibility criteria**

Name of the rater:

Date of rating:

Title of the paper:

Author:

Year of publication:

Name of the journal:

| Study Characteristics | Eligibility criteria  *(Insert inclusion criteria for each characteristic as defined in the Protocol)* | | Eligibility criteria met? | | |
| --- | --- | --- | --- | --- | --- |
|  |  |  | Yes | No | Unclear |
| Type of study | Randomised Controlled Trial (crossover or parallel design) | |  |  |  |
| Participants | Diagnosed with ASD | |  |  |  |
| Types of intervention | Mood stabilisers (either lithium or anti-epileptics) | |  |  |  |
| Types of comparison | Placebo or another intervention | |  |  |  |
| Types of outcome measures | ASD core symptoms and/or other associated behavioural or psychiatric symptoms | |  |  |  |
| Number of participants | Ten or more | |  |  |  |
| INCLUDE | | EXCLUDE UNCERTAIN | | | |
| Reason for exclusion |  | | | | |
| Notes: Get full paper if uncertain or for inclusion | | | | | |

**Appendix 3: Data extraction proforma** (adapted from Cochrane Collaboration)

Notes on using data extraction form:

- Be consistent in the order and style you use to describe the information for each report.
- Record any missing information as unclear or not described, to make it clear that the information was not found in the study report(s), not that you forgot to extract it.
- Include any instructions and decision rules on the data collection form, or in an accompanying document. It is important to practice using the form and give training to any other authors using the form.

Title of the systematic review:

**General Information**

| Date form completed *(dd/mm/yyyy)* |  |
| --- | --- |
| Name/ID of person extracting data |  |
| Reference citation (full citation) |  |
| Study author contact details (Email) |  |
| Publication type *(e.g., full report, abstract, letter)* |  |
| Notes: | |

Characteristics of the included study

**Participants**

|  | Description  *Include comparative information for each intervention or comparison group if available* | |
| --- | --- | --- |
| Population description *(from which study participants are drawn)* |  | |
| Setting *(e.g., intensive care unit, service providers, institutions, day care centre etc)* |  | |
| Method of recruitment of participants *(e.g., phone, mail, clinic patients)* |  | |
| Informed consent obtained | Yes No Unclear |  |
| Intervention group | Age of participants (range, mean & SD) |  |
|  | Number (%) of participants by gender |  |
|  | Number (%) with ID, ADHD or other NDDs |  |
|  | Type of pharmacological regime (antiepileptics/ lithium) + name of medication + dose |  |
|  | Co morbidity (psychiatric) |  |
|  | Co morbidity (physical) |  |
|  | Adverse events (number and %) |  |
| Control group | Age of participants (range, mean & SD) |  |
|  | Number (%) of participants by gender |  |
|  | Number (%) with ID, ADHD or other NDDs |  |
|  | Type of intervention (placebo or another medication or other intervention) + name + dose |  |
|  | Co morbidity (psychiatric) |  |
|  | Co morbidity (physical) |  |
|  | Adverse events (number and %) |  |

**Methods**

|  | Descriptions as stated in report/paper | Location in text or source *(pg & /fig/table/other)* |
| --- | --- | --- |
| Aim of study *(e.g., efficacy, equivalence, pragmatic)* |  |  |
| Design *(e.g., parallel, crossover)* |  |  |
| Sampling technique (e.g., random) |  |  |
| Method of establishing ASD diagnosis (if known) (clinical or ICD or DSM or ADI-R or ADOS etc.) |  |  |

**Outcomes**

*Copy and paste table for each outcome.*

**Outcome 1**

|  | Description as stated in report/paper | | | | | | | Location in text or source *(pg & /fig/table/other)* |
| --- | --- | --- | --- | --- | --- | --- | --- | --- |
| Primary outcome if dichotomous (e.g. %) (name the outcome and the instrument used to measure the outcome) | Number (%) in the intervention arm | Total number of participants in the intervention arm | | Number (%) in the control arm | | | Total number of participants in the control arm |  |
|  |  |  | |  | | |  |  |
| Primary outcome if continuous | Mean in the intervention arm | SD in the intervention arm | Mean in the control arm | | | SD in the control arm | |  |
|  |  |  |  | | |  | |  |
| Duration of intervention (weeks/months) (if crossover, add duration of baseline and washout period) |  | | | | | | |  |
| Duration of follow up (weeks/months) |  | | | | | | |  |
| Statistical methods used and appropriateness of these *(e.g., proportion, %, risk ratio, odds ratio)* |  | | | | | | |  |
| Secondary outcomes |  | | | | | | |  |
| Number of missing data |  | | | | | | |  |
| Reason for missing data |  | | | | | | |  |
| Other |  | | | | | | |  |
| Is outcome/tool validated? | Yes No Unclear | | | | Name of the tool: | | |  |
| Notes: | | | | | | | | |

**Other information**

|  | Description as stated in report/paper | Location in text or source *(pg & /fig/table/other)* |
| --- | --- | --- |
| Main findings (statistically significant difference or not; provide P value or other relevant data in support of main findings (primary and secondary outcomes) |  |  |
| Key conclusions of study authors |  |  |
| Your critique of the study (any design flaw etc.) |  |  |
| Your own overall conclusion |  |  |
| Correspondence required for further study information *(from whom, what and when)* |  | |
| Notes: | | |

**Other**

| Study funding sources *(including role of funders)* |  |  |
| --- | --- | --- |
| Possible conflicts of interest *(for study authors)* |  |  |
| Notes: | | |

**Appendix 4**: **Cochrane Risk of Bias proforma**

*See* [*Chapter 8*](http://www.mrc-bsu.cam.ac.uk/cochrane/handbook/index.htm#chapter_8/8_assessing_risk_of_bias_in_included_studies.htm) *of the Cochrane Handbook. Additional domains may be added for non-randomised studies.*

| Domain | Risk of bias | | | Support for judgement  *(include direct quotes where available with explanatory comments)* | Location in text or source *(pg & /fig/table/other)* |
| --- | --- | --- | --- | --- | --- |
|  | Low | High | Unclear |  |  |
| Random sequence generation  *(selection bias)* |  |  |  |  |  |
| Allocation concealment  *(selection bias)* |  |  |  |  |  |
| Blinding of participants and personnel  *(performance bias)* |  |  |  | Outcome group: All/ |  |
| *(if separate judgement by outcome(s) required)* |  |  |  | Outcome group: |  |
| Blinding of outcome assessment  *(detection bias)* |  |  |  | Outcome group: All/ |  |
| *(if separate judgement by outcome(s) required)* |  |  |  | Outcome group: |  |
| Incomplete outcome data  *(attrition bias)* |  |  |  | Outcome group: All/ |  |
| *(if separate judgement by outcome(s) required)* |  |  |  | Outcome group: |  |
| Selective outcome reporting?  *(reporting bias)* |  |  |  |  |  |
| Other bias |  |  |  |  |  |
| Notes: | | | | | |

**Appendix 5: Funnel plot OAS/OAS-M, CGI-I and ABC-I**

Funnel plot OAS/OAS-M


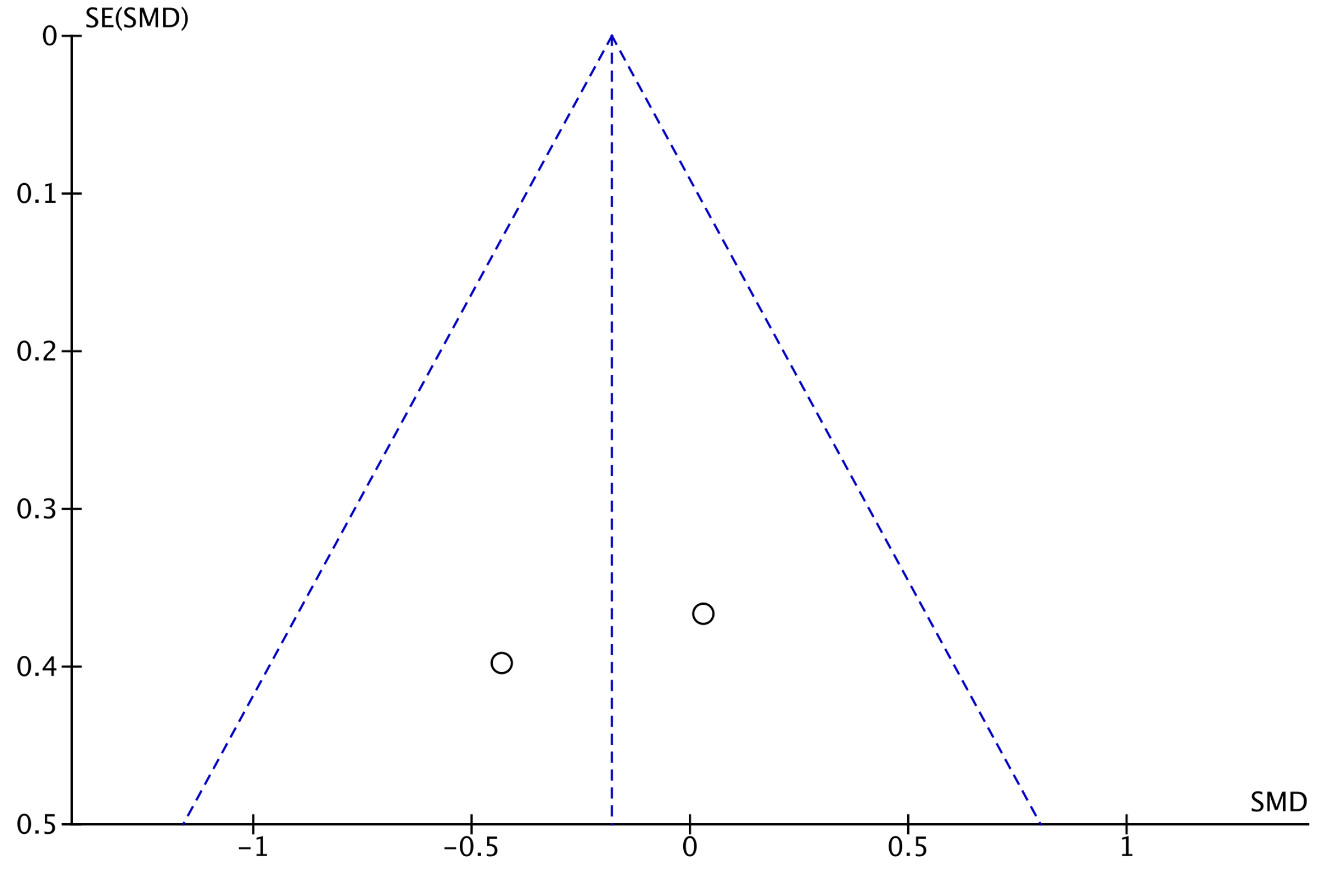


Funnel plot CGI-I


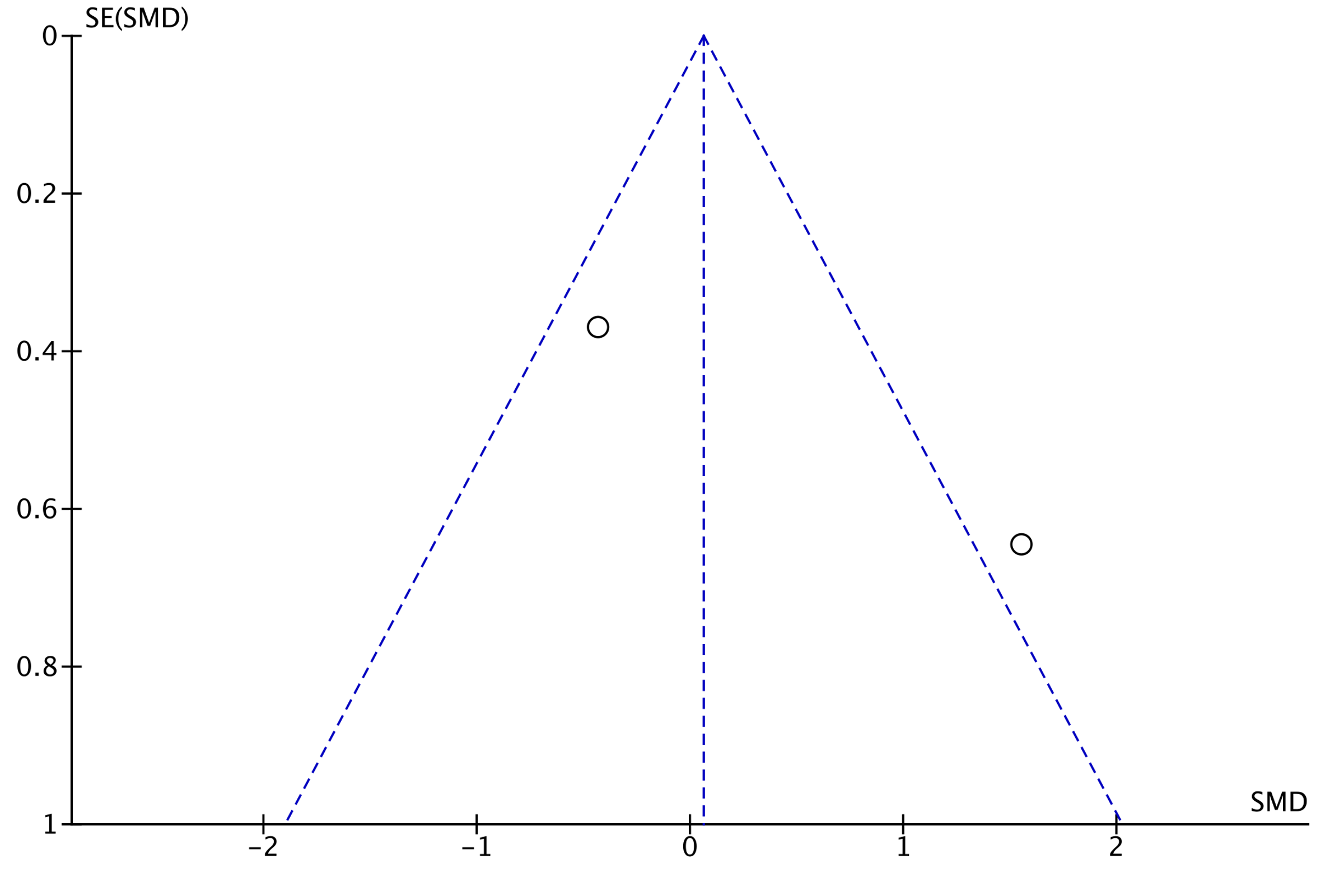


Funnel plot ABC-I


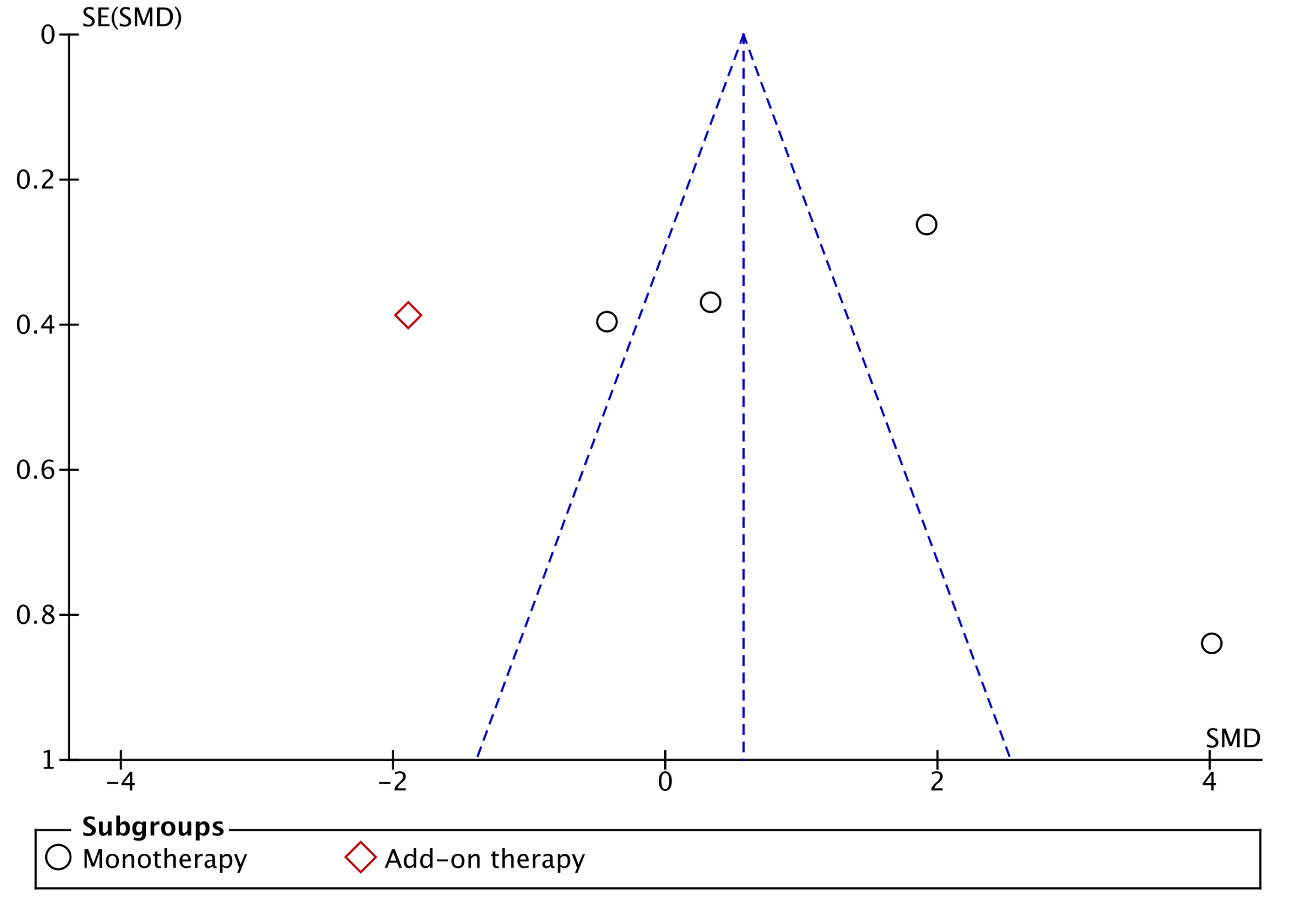


**Appendix 6**: **AMSTAR 2 checklist**

| 1. Did the research questions and inclusion criteria for the review include the components of PICO? | | | | | |
| --- | --- | --- | --- | --- | --- |
| For Yes:   - Population - Intervention - Comparator group - Outcome | | Optional (recommended)   Timeframe for follow-up | x   | Yes No |  |
| **2. Did the report of the review contain an explicit statement that the review methods were established prior to the conduct of the review and did the report justify any significant deviations from the protocol?** | | | | | |
|  | For Partial Yes:  The authors state that they had a written protocol or guide that included ALL the following:   - review question(s) - a search strategy - inclusion/exclusion criteria - a risk of bias assessment | For Yes:  As for partial yes, plus the protocol should be registered and should also have specified:   - a meta-analysis/synthesis plan, if appropriate, *and* - a plan for investigating causes of heterogeneity - justification for any deviations from the protocol | x     | Yes Partial Yes No |  |
| **3. Did the review authors explain their selection of the study designs for inclusion in the review?** | | | | | |
|  | For Yes, the review should satisfy ONE of the following:   - *Explanation for* including only RCTs - OR *Explanation for* including only NRSI - OR *Explanation for* including both RCTs and NRSI | |  | Yes |  |
| **4. Did the review authors use a comprehensive literature search strategy?** | | | | | |
|  | For Partial Yes (all the following): | For Yes, should also have (all the following):   - searched the reference lists / bibliographies of included studies - searched trial/study registries - included/consulted content experts in the field - where relevant, searched for grey literature - conducted search within 24 months of completion of the review |  |  |  |
|  | - searched at least 2 databases (relevant to research question) - provided key word and/or search strategy - justified publication restrictions |  |   x   | Yes Partial Yes No |  |
|  | (e.g. language) |  |  |  |  |
|  | **5. Did the review authors perform study selection in duplicate?** | |  | |  |
|  | For Yes, either ONE of the following:   - at least two reviewers independently agreed on selection of eligible studies and achieved consensus on which studies to include - OR two reviewers selected a sample of eligible studies and achieved good agreement (at least 80 percent), with the remainder selected by one reviewer. | | x   | Yes No |  |

| **6. Did the review authors perform data extraction in duplicate?** | | | | | | | |
| --- | --- | --- | --- | --- | --- | --- | --- |
| For Yes, either ONE of the following:   - at least two reviewers achieved consensus on which data to extract from included studies - OR two reviewers extracted data from a sample of eligible studies and achieved good agreement (at least 80 percent), with the remainder extracted by one reviewer. | | | | | | x Yes   - No | |
| **7. Did the review authors provide a list of excluded studies and justify the exclusions?** | | | | | | | |
|  | | For Partial Yes:   provided a list of all potentially relevant studies that were read  in full-text form but excluded from the review | For Yes, must also have:   Justified the exclusion from the review of each potentially relevant study | | | x Yes   - Partial Yes - No | |
| **8. Did the review authors describe the included studies in adequate detail?** | | | | | | | |
|  | | For Partial Yes (ALL the following):   - described populations - described interventions - described comparators - described outcomes - described research designs | For Yes, should also have ALL the following:   - described population in detail - described intervention in detail (including doses where relevant) - described comparator in detail (including doses where relevant) - described study’s setting - timeframe for follow-up | | | x Yes   - Partial Yes - No | |
| **9. Did the review authors use a satisfactory technique for assessing the risk of bias (RoB) in individual studies that were included in the review?** | | | | | | | |
|  | | **RCTs**  For Partial Yes, must have assessed RoB from   - unconcealed allocation, *and* - lack of blinding of patients and assessors when assessing outcomes (unnecessary for objective outcomes such as all-   cause mortality) | For Yes, must also have assessed RoB from:   - allocation sequence that was not truly random, *and* - selection of the reported result from among multiple measurements or analyses of a specified outcome | | | x Yes   - Partial Yes - No - Includes only NRSI | |
|  | | **NRSI**  For Partial Yes, must have assessed RoB:   - from confounding, *and* - from selection bias   **10. Did the review authors report o** | For Yes, must also have assessed RoB:   - methods used to ascertain exposures and outcomes, *and* - selection of the reported result from among multiple measurements or analyses of a specified outcome   **n the sources of funding for the studies inc** | | | - Yes - Partial Yes - No - Includes only RCTs   **luded in the review?** | |
|  | | For Yes   Must have reported on the sources of funding for individual studies included x Yes in the review. Note: Reporting that the reviewers looked for this information  No but it was not reported by study authors also qualifies | | | | | |
| **11. If meta-analysis was performed did the review authors use appropriate methods for statistical combination of results?** | | | | | | | |
|  | **RCTs**  For Yes:   - The authors justified combining the data in a meta-analysis   - AND they used an appropriate weighted technique to combine study results and adjusted for heterogeneity if present.   - AND investigated the causes of any heterogeneity | | | | x Yes   - No - No meta-analysis conducted | |  |
|  | **For NRSI**  For Yes:   - The authors justified combining the data in a meta-analysis   - AND they used an appropriate weighted technique to combine study results, adjusting for heterogeneity if present   - AND they statistically combined effect estimates from NRSI that were adjusted for confounding, rather than combining raw data, or justified combining raw data when adjusted effect estimates were not available   - AND they reported separate summary estimates for RCTs and NRSI separately when both were included in the review | | | | - Yes - No - No meta-analysis conducted | |  |
| **12. If meta-analysis was performed, did the review authors assess the potential impact of RoB in individual studies on the results of the meta-analysis or other evidence synthesis?** | | | | | | | |
|  | For Yes:   - included only low risk of bias RCTs - OR, if the pooled estimate was based on RCTs and/or NRSI at variable RoB, the authors performed analyses to investigate possible impact of RoB on summary estimates of effect. | | | | x Yes   - No - No meta-analysis conducted | |  |
| **13. Did the review authors account for RoB in individual studies when interpreting/ discussing the results of the review?** | | | | | | | |
|  | For Yes:   - included only low risk of bias RCTs - OR, if RCTs with moderate or high RoB, or NRSI were included the review provided a discussion of the likely impact of RoB on the results | | | | x Yes   - No | |  |
| **14. Did the review authors provide a satisfactory explanation for, and discussion of, any heterogeneity observed in the results of the review?** | | | | | | | |
|  | For Yes:   - There was no significant heterogeneity in the results - OR if heterogeneity was present the authors performed an investigation of sources of any heterogeneity in the results and discussed the impact of this on the results of the review | | | | x Yes   - No | |  |
| **15. If they performed quantitative synthesis did the review authors carry out an adequate investigation of publication bias (small study bias) and discuss its likely impact on the results of the review?** | | | | | | | |
|  | For Yes:   performed graphical or statistical tests for publication bias and discussed the likelihood and magnitude of impact of publication bias | | | | x Yes   - No - No meta-analysis conducted | |  |
| **16. Did the review authors report any potential sources of conflict of interest, including any funding they received for conducting the review?** | | | | | | | |
|  | For Yes:   - The authors reported no competing interests OR - The authors described their funding sources and how they managed potential conflicts of interest | | | x Yes   - No | | |  |
